# Correlation and Comparison of Cortical and Hippocampal Neural Progenitor Morphology and Differentiation through the Use of Micro- and Nano-Topographies

**DOI:** 10.3390/jfb8030035

**Published:** 2017-08-12

**Authors:** Sharvari Sathe, Xiang Quan Chan, Jing Jin, Erik Bernitt, Hans-Günther Döbereiner, Evelyn K.F. Yim

**Affiliations:** 1 Mechanobiology Institute, National University of Singapore, T-Lab, #05-01, 5A Engineering Drive 1, Singapore 117411; sharvari.sathe@u.nus.edu; hgd@biophysik.uni-bremen.de (H.-G.D.); 2 Department of Biomedical Engineering, National University of Singapore, 4 Engineering Drive 3, Engineering Block 4, #04-08, Singapore 117583; xiangquan.c@gmail.com (X.Q.C.); jing.jin0925@gmail.com (J.J.); 3 Institut für Biophysik, Universität Bremen, Otto-Hahn-Allee 1, Bremen 28359, Germany; erik-bernitt@web.de (E.B.); 4 Department of Surgery, Yong Loo Lin School of Medicine, National University of Singapore, NUHS Tower Block, Level 8, 1E Kent Ridge Road, Singapore 119228; 5 Department of Chemical Engineering, University of Waterloo, 200 University Avenue West, Waterloo, ON N2L 3G1, Canada

**Keywords:** neuronal differentiation, topography, cell morphology, neural progenitor cells, neurons, astrocytes, gratings, polydimethlysiloxane, correlation analysis, quantitative comparison

## Abstract

Neuronal morphology and differentiation have been extensively studied on topography. The differentiation potential of neural progenitors has been shown to be influenced by brain region, developmental stage, and time in culture. However, the neurogenecity and morphology of different neural progenitors in response to topography have not been quantitatively compared. In this study, the correlation between the morphology and differentiation of hippocampal and cortical neural progenitor cells was explored. The morphology of differentiated neural progenitors was quantified on an array of topographies. In spite of topographical contact guidance, cell morphology was observed to be under the influence of regional priming, even after differentiation. This influence of regional priming was further reflected in the correlations between the morphological properties and the differentiation efficiency of the cells. For example, neuronal differentiation efficiency of cortical neural progenitors showed a negative correlation with the number of neurites per neuron, but hippocampal neural progenitors showed a positive correlation. Correlations of morphological parameters and differentiation were further enhanced on gratings, which are known to promote neuronal differentiation. Thus, the neurogenecity and morphology of neural progenitors is highly responsive to certain topographies and is committed early on in development.

## 1. Introduction

Establishment of cellular morphology is one of the first processes in neurons that has been described to be affected by topography through contact guidance [1]. Neurons establish cell morphology in vitro after attachment to a substrate, which initiates the process of neurite guidance and extension [2]. During differentiation, neural progenitor cells (NPCs) form neurons, astrocytes, and oligodendrocytes [3] with distinct morphologies. Multiple biochemical pathways come into play during the transition of a cell from a progenitor state to a differentiated state. At the mechanical level, cues, such as substrate topography, are important determinants in the differentiation of multipotent cells into either neurons or astrocytes [4]. However, it has been a challenge to conduct a quantitative morphometric analysis of the progenitor response to varying topographical cues. 

Firstly, the tracing of neurons or astrocytes on unpatterned substrates needed to shift from a manual process to a largely automated process in order to have a statistical understanding of morphology. This has been achieved with the creation of high-throughput screening tools, specifically for neurons, such as NeuronJ [5], Metamorph [6], algorithms developed in MATLAB [7,8], etc. Secondly, there have been few systematic analyses of topographical influences on differentiation conducted on an array of topographies [9,10,11]. Most studies vary one topographical feature to assess the possible mechanism underlying observable cell shape changes [4]. However, none of these studies have directly compared the response of progenitors from different origins when exposed to an array of topographical cues. Intrinsic differences exist in the neurogenecity of neural progenitors of different origins depending on the species of origin, the region of the brain, and the progenitor age at which the NPCs are isolated [12,13]. Thus, we hypothesized that the morphological changes of NPCs differentiated on topographies would be influenced by their origin. 

In the present study, we aim to understand the complex relationship between cell morphology and differentiation in cortical and hippocampal murine NPCs (mNPCs) with the use of an array of 14 topographies. The following four morphological parameters were measured: the length of the longest extension, the number of extensions, the number of branches, and the soma area of both neurons and astrocytes derived from mNPCs. These parameters are known to be hallmarks of the impact of biochemical and mechanical perturbations on neuronal cells. For example, genetic mutations introduced into dopaminergic neurons have been shown to cause a reduction in neurite length and branching [14]. Rett Syndrome brains (*MECP2* gene knockouts) are known to show decreased neuronal size and synapse elimination in vitro [15]. In addition, exposure to neurite outgrowth inhibiting chemicals at various concentrations, has been reported to cause drastic increases and decreases in neurite extension in high-content screening assays [16]. Finally, topography has been shown to be one of the biggest modulators of cellular morphology, especially in size ranges matching specific organelles of interest [4].

The relation of topography with differentiation efficiency has been vastly explored. Studies with neural progenitors have been conducted on surfaces with features such as gratings, pillars, wells (reviewed in [17]), or even recently, hierarchical structures [11,18]. Thus, how differentiation and morphology correlate with each other remains an important topic of discussion in numerous studies of neuronal differentiation on topographical substrates [4]. This study systematically correlates the morphological changes caused by topographical cues with the differentiation efficiency of neural progenitors from different origins.

## 2. Results

### 2.1. Neural Progenitors from Different Origins Show Inherent Morphological Differences

**Figure 1:**
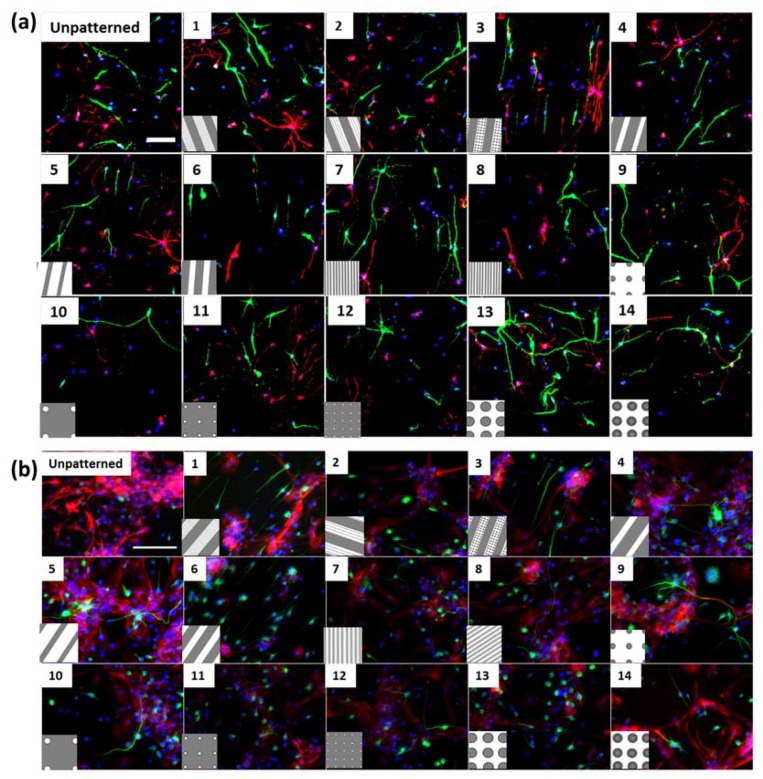
Neurons and astrocytes show a range of morphologies on different topographies and also show inherent differences based on progenitor source: (**a**) immunofluorescence images of cortical mNPC-derived neurons and astrocytes on the 14 topographies. Scale bar = 50 µm; (**b**) immunofluorescence images (from [10] with permission from John Wiley and Sons) of hippocampal mNPC-derived neurons and astrocytes on the 14 topographies. Scale bar = 50 µm. Green: neurons, red: astrocytes, blue: nuclei. Topographies are listed in Table 1. Boxes show schematic representation of underlying topography and its alignment (not to scale). Representative phase contrast images of differentiated cells on topography are presented in Figure S1i–l.

Cortical and hippocampal neural progenitor cells were differentiated on 14 topographical patterns (Table 1). These patterns served as modulators of morphology. The mNPC-derived neurons were identified by immunofluorescence staining with the TUJ1 antibody (green), and mNPC-derived astrocytes were identified with GFAP antibody (red) (Figure 1). 

The source of the NPCs played a role in the extent to which topography could modulate the morphology of the differentiated cells. The immunofluorescence staining in Figure 1 shows the qualitative differences observed in the cells differentiated on the various patterns, as well as the unpatterned control. Comparing the two cell types revealed that the cell morphology was differently affected by the same topographies. The morphology of neurons appeared similar on unpatterned substrates for both types of differentiated NPCs, but the astrocytes showed inherent differences in the number of projections and soma areas. With respect to topographical influence, alignment to anisotropic topographies is indicative of the perception of the cells to their mechanical microenvironment. In the present study, both types of neurons showed alignment to gratings substrates (Patterns 1–8) but with different degrees of alignment (Figure 1 and phase contrast images in Figure S1). For example, in the representative images, cortical mNPC-derived neurons on Patterns 3–7 showed alignment with the underlying gratings, whereas hippocampal mNPC-derived neurons showed alignment on Patterns 1, 2, and 6. Astrocyte alignment to topographies was different for the two types of cells. The cortical mNPC-derived astrocytes appear to be aligned on the same topographies as those described for neurons, but the hippocampal mNPC-derived astrocytes did not show alignment. Although the morphologies of the two cell types were further diversified due to the topographical effects, the response to the topographies shown by the cortical and hippocampal mNPCs corresponded to the origin of the cells.

### 2.2. Pillar Topographies Show Higher Extension Lengths in Differentiated Hippocampal mNPCs

**Figure 2:**
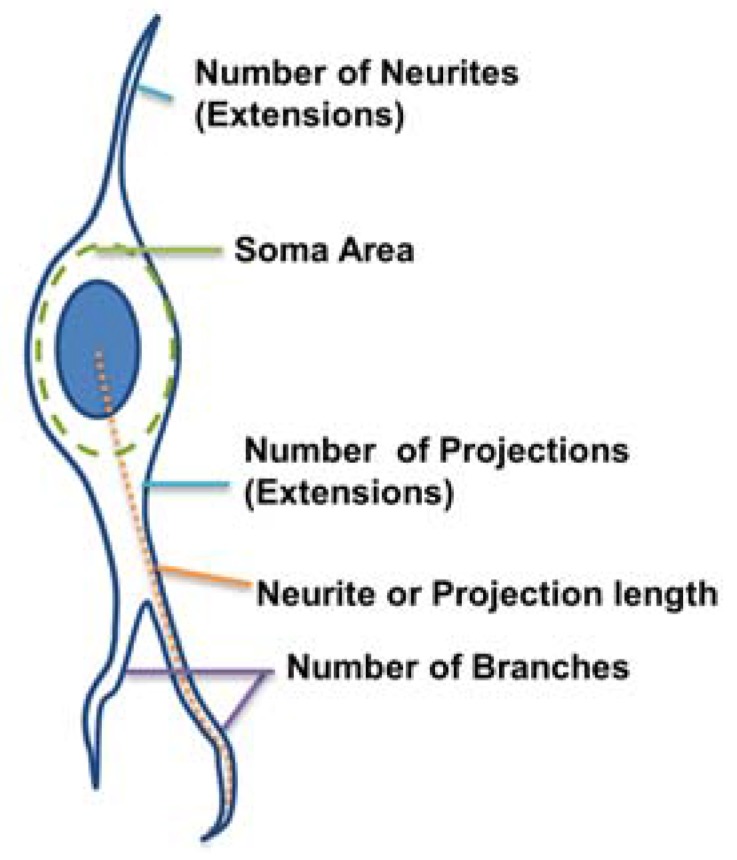
Morphological parameters of interest: the number of extensions (neurites for neurons or projections for astrocytes) from the cell center, the soma area (the area of the main cell body), the extension length, and the number of branches per extension.

In order to elucidate the observed morphological differences between differentiated hippocampal and cortical mNPCs, morphological parameters of the cells (length of the longest extension, number of extensions, number of branches, and soma area) were quantified on each of the topographies (Figure 2). 

**Figure 3:**
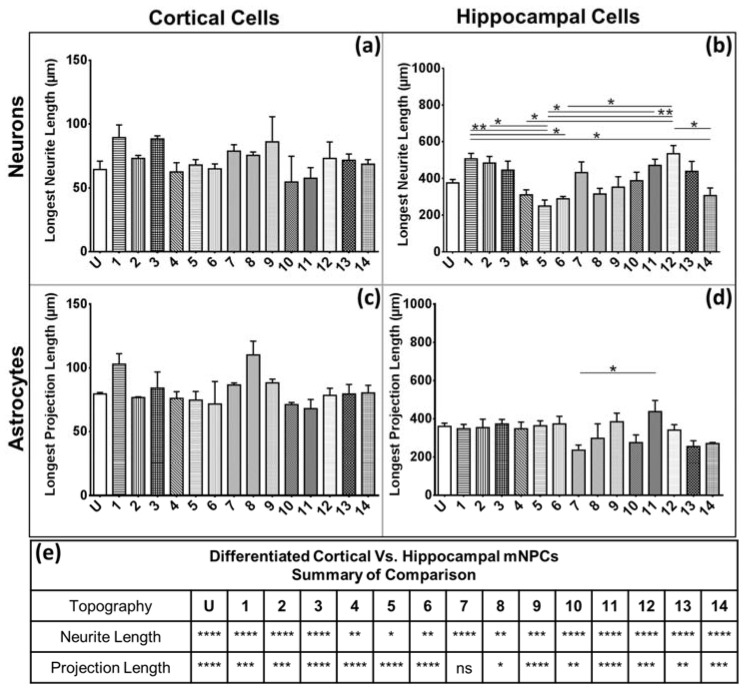
Hippocampal cells show longer extension lengths. Graphs depict average length of the longest neurite per neuron derived (**a**) cortical NPCs; (**b**) hippocampal NPCs; and the average length of the longest projection per astrocyte derived from (**c**) cortical NPCs and (**d**) hippocampal NPCs; one-way ANOVA: * *p* < 0.05, ** *p* < 0.01; (**e**) comparison of extension lengths of differentiated cortical and hippocampal mNPCs on the same topographies. Two-way ANOVA: * *p* < 0.05, ** *p* < 0.01, *** *p* < 0.001, **** *p* < 0.0001, ns: no significant difference. All data are represented as data ± SEM, *N* = 3 biological replicates.

Extension length was defined as the path length from the cell center to the end of the longest extension on that cell. The extension lengths were measured and then averaged for each population of cells. Although cortical mNPC-derived neurons showed no differences in neurite length on different topographies (Figure 3a), hippocampal mNPC-derived neurons showed statistically significant differences (Figure 3b). Neurite lengths were the longest on Pattern 12 (pillars with 130 nm diameter, 400 nm pitch, 230 nm height), which were significantly higher than those measured on microgratings without hierarchical structures (Patterns 4–6) and Pattern 14 (1.8 μm diameter microlenses). Pattern 5 (2 μm gratings with 1 μm spacing and 80 nm height) generated neurons with the shortest neurite lengths (2.5 ± 0.3 × 10^2^ μm), significantly less than Pattern 1 (2 μm gratings with perpendicular hierarchical 250 nm gratings) (*p* < 0.01), Pattern 2 (2 μm gratings with parallel hierarchical 250 nm gratings) (*p* < 0.05), Pattern 11 (500 nm diameter pillars) (*p* < 0.05), and Pattern 12 (130 nm pillars) (*p* < 0.01). Therefore, gratings topographies (Patterns 1–8) showed variation in the neurite lengths of the differentiated hippocampal NPCs. Overall, the pillar topographies studied (micro and nano ranges) caused an increase in neurite length of hippocampal cells. 

Cortical mNPC-derived astrocytes did not show any trends on different topographies (Figure 3c). Hippocampal mNPC-derived astrocytes were observed to show spongiform morphology (Figure 1b, red). Extension length on these cells was defined as the path length from the cell center to the end of the longest protrusion from the soma. In general, hippocampal mNPC-derived astrocytes showed significantly longer projection lengths (3.4 ± 0.6 × 10^2^ µm) as compared to cortical mNPC-derived astrocytes (Figure 3c–e).

Thus, the extension length of differentiated cortical mNPCs was not significantly influenced by topographies, whereas hippocampal NPC-derived neurons seemed more responsive to certain substrates, such as pillars and gratings. In addition, the average extension length of differentiated hippocampal mNPCs (366 ± 8 µm) was significantly longer than that of differentiated cortical mNPCs (77 ± 1 µm) (Figure 3e). 

### 2.3. Cortical mNPC-derived Cells Show More Extensions Than Differentiated Hippocampal Cells

The number of extensions possessed by neurons is indicative of their connectivity to surrounding cells. Therefore, in addition to the length of the extensions, it was of interest to quantify the number of extensions present on the differentiated cells. The average number of extensions per cell was calculated as the number of extensions each cell possessed divided by the total number of cells. In some cases, the majority of neurons and astrocytes were round and did not show any extensions from the cell center. In such cases, the average number of extensions possessed by the population was less than 1. 

**Figure 4:**
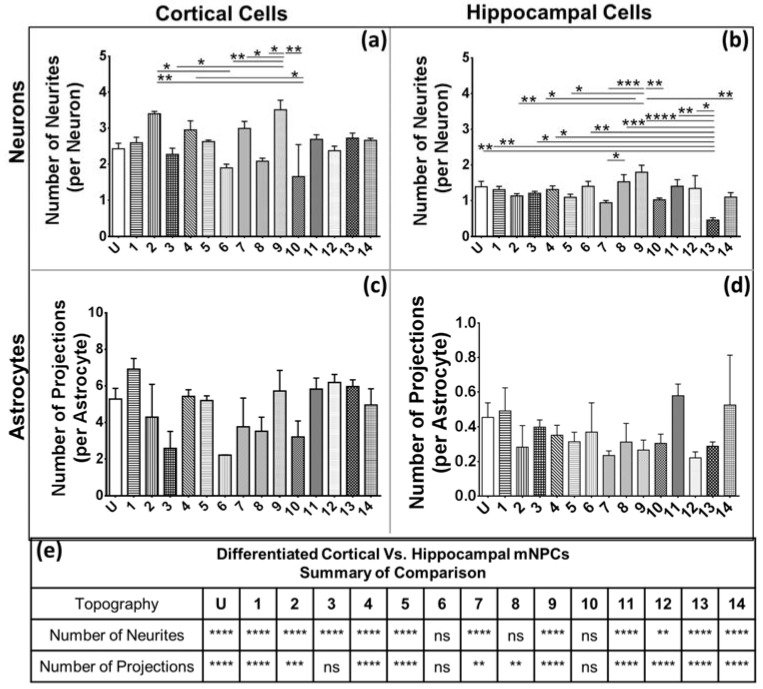
Cortical mNPC-derived astrocytes show a high number of extensions from the cell center: graphs depict average number of neurites per neuron derived from (**a**) cortical NPCs; (**b**) hippocampal NPCs; and average number of projections per astrocyte derived from (**c**) cortical NPCs and (**d**) hippocampal NPCs. One-way ANOVA: * *p* < 0.05, ** *p* < 0.01, *** *p* < 0.001, **** *p* < 0.0001; (**e**) Comparison of differentiated cortical and hippocampal mNPCs on the same topographies with respect to the number of neurites per neuron and projections per astrocyte. Two-way ANOVA: * *p* < 0.05, ** *p* < 0.01, *** *p* < 0.001, **** *p* < 0.0001, ns- no significant difference. All data are represented as data ± SEM, *N* = 3 biological replicates.

The average number of neurites on cortical and hippocampal mNPC-derived neurons was less than 4 (Figure 4). Although cortical mNPC-derived neurons showed shorter projections, as seen previously (Figure 3a,b), they possessed a significantly higher number of projections than hippocampal mNPC-derived neurons on most topographies (Figure 4e). Cortical mNPC-derived neurons had an average of 2 neurites extending from the center of the cell per neuron. Pattern 9 (1 μm wells) and Pattern 2 (2 μm gratings with parallel 250 nm gratings) showed cells with the largest number of neurites per neuron (>3), while Pattern 10 (2 μm diameter pillars with 12 μm pitch and 2 μm height) showed neurons with the least number of neurites. On the other hand, differentiated hippocampal cells had, on average, 1 neurite extending from the center per neuron (Figure 2b,d). Amongst all topographies, neurons derived from both types of mNPCs on Pattern 9 (1 μm wells) showed the highest number of projections (Figure 4a,b). The 1 μm wells generated neurons with the largest number of neurites amongst the hippocampal mNPC-derived neurons, which were significantly higher than those on Patterns 2, 3, 5, 7, 10, 13, and 14. Pattern 13 (1 μm microlenses) showed neurons with the smallest number of neurites, with an average of 0.5 neurites per neuron. This number is significantly smaller than most other patterns and has the most significant difference in comparison to Pattern 9 (1 μm wells) (*p* < 0.0001). Thus, differentiation of cells on Pattern 9 (1 μm wells) was associated with an increase in the number of neurites expressed by both cortical and hippocampal NPC derived neurons.

Cortical mNPC-derived astrocytes showed a large degree of variation in the number of projections from the center (Figure 4c), but showed a significantly higher number of projections per astrocyte than hippocampal mNPC-derived astrocytes (Figure 4e). Hippocampal mNPC-derived astrocytes showed an average of less than 0.5 extensions from the cell center, significantly less than the cortical mNPC-derived astrocytes (Figure 4e). The average number of extensions from the cell center being less than 1 is indicative that most cells did not show any extensions from the cell center.

### 2.4. Branching Shows a Large Degree of Variation on Topography

Branching is another indicator of connectivity of cells. A branch was defined as a secondary protrusion from an extension originating from the cell center (Figure 2). Thus, each extension could possess multiple branches. When an extension from the cell center displayed no secondary protrusion, the neurite was counted as possessing zero branches. 

**Figure 5:**
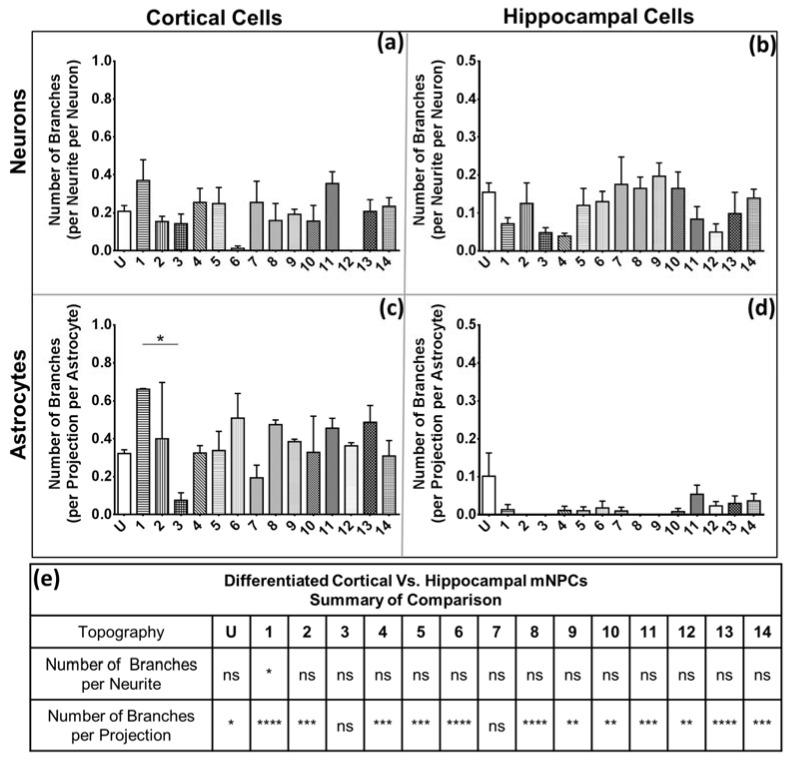
Hippocampal mNPC-derived cells show fewer branches per extension than cortical mNPC-derived cells. Graphs depict average branches per neurite per neuron derived from (**a**) cortical NPCs with topography 12 showing no branches on neuritis; (**b**) hippocampal NPCs; and average branches per projection per astrocyte derived from (**c**) cortical NPCs and (**d**) hippocampal NPC. One-way ANOVA: * *p* < 0.05, ** *p* < 0.01, *** *p* < 0.001, **** *p* < 0.0001; (**e**) comparison of the number of branches per neurite per neuron and the number of branches per projection per astrocyte on the same topographies derived from cortical and hippocampal mNPCs. Two-way ANOVA: * *p* < 0.05, ** *p* < 0.01, *** *p* < 0.001, **** *p* < 0.0001, ns- no significant difference. All data are represented as data ± SEM, *N* = 3 biological replicates.

Almost all the differentiated neurons displayed a lack of branching on the extended neurites; hence, cells often displayed neurites with no protrusions (Figure 5a,b). Neurons displayed different morphological effects of the topography depending on the mNPC type that they were derived from. In cortical mNPC-derived neurons, Pattern 1 (2 μm gratings with perpendicular 250 nm gratings) showed the most number of branches, with an average of 0.4 ± 0.1 branch per neurite per neuron (Figure 5a). This suggests that most neurites did not possess branches. Similarly, hippocampal mNPC-derived neurons showed a maximum of 0.2 ± 0.07 branches per neurite on Pattern 9 (1 μm wells) (Figure 5b). Therefore, most neurons did not display any branches on their neurites, resulting in the average number of branches being less than 1.

Cortical mNPC-derived astrocytes showed significantly more branches than hippocampal mNPC-derived astrocytes (Figure 5e). The highest average number of branches on cortical mNPC-derived astrocytes was 0.662 ± 0.003 on the 2 μm gratings with perpendicular 250 nm gratings (Figure 5c). It was observed that topographical substrates reduced the branching on hippocampal mNPC-derived astrocytes (Figure 5d). Unpatterned substrates showed the most branching (0.10 ± 0.06), whereas multiple topographies displayed no branches at all. Thus, the degree of branching on projections was largely influenced by the type of NPC from which astrocytes were differentiated.

### 2.5. Hippocampal mNPC-derived Astrocytes Show Large Soma Areas

The soma area of neural cells has been associated with neurological disease [19]. Consequently, the quantification of the soma areas of the differentiated NPCs proved to be of relevance. The soma area was defined as the area of the cell excluding the extensions from the cell body (Figure 2).

**Figure 6:**
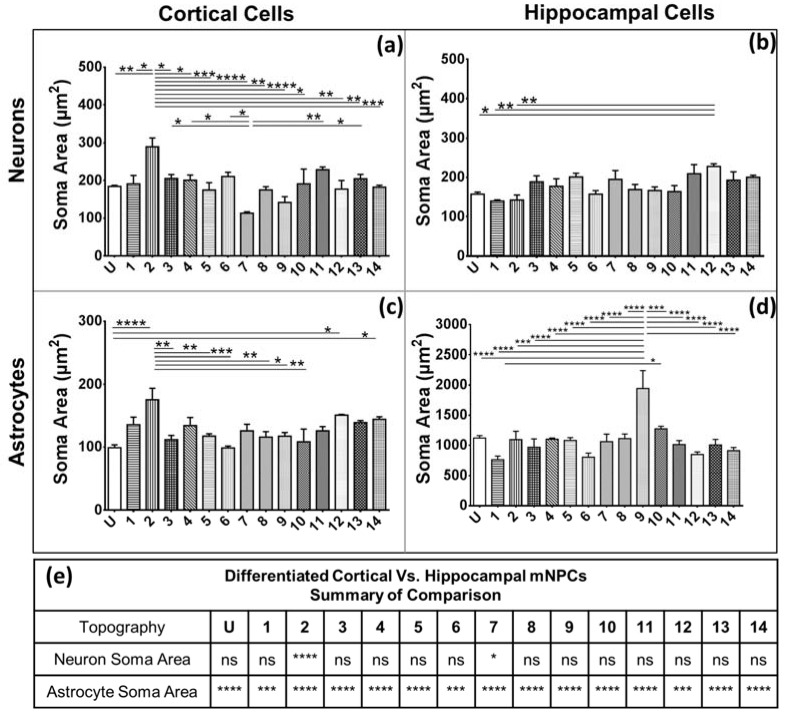
Hippocampal mNPC-derived astrocytes have very large soma areas. Graphs depict average soma areas for neurons derived from (**a**) cortical NPCs; (**b**) hippocampal NPCs; and for astrocytes derived from (**c**) cortical NPCs and (**d**) hippocampal NPCs. One-way ANOVA: * *p* < 0.05, ** *p* < 0.01, *** *p* < 0.001, **** *p* < 0.0001. (**e**) Comparison of the soma areas of neurons and astrocytes on the same topographies derived from cortical and hippocampal mNPCs. Two-way ANOVA: * *p* < 0.05, ** *p* < 0.01, *** *p* < 0.001, **** *p* < 0.0001, ns: no significant difference. All data are represented as data ± SEM, *N* = 3 biological replicates.

The average cortical mNPC-derived neuron soma area was greater than 200 μm^2^ (Figure 6a). Pattern 2 (2 μm gratings with parallel 250 nm gratings) generated neurons with the largest soma areas of 2.9 ± 0.2 × 10^2^ μm^2^. The soma of neurons on Pattern 2 were significantly larger than almost all other topographies, with the most significant differences occurring with Patterns 7 and 9 (*p* < 0.0001). Hippocampal mNPC-derived neurons showed average soma areas close to 180 μm^2^. Pattern 12 showed the largest neuron areas (2.28 ± 0.07 × 10^2^ μm^2^); while Pattern 1 showed the smallest neurons (Figure 6b). Cortical mNPC-derived astrocytes had significantly smaller soma areas (1.3 ± 0.2 × 10^2^ μm^2^) than hippocampal mNPC-derived astrocytes (1.1 ± 0.3 × 10^3^ μm^2^) (Figure 6e). The largest soma areas on cortical mNPC-derived astrocytes were generated on Pattern 2 (2 μm gratings with parallel 250 nm gratings) (1.8 ± 0.2 × 10^2^ μm^2^) which were significantly larger than the soma areas on multiple other patterns (Figure 6c). 

Hippocampal mNPC-derived astrocytes showed large soma areas on all substrates in comparison to cortical mNPC-derived cells and hippocampal mNPC-derived neurons (Figure 6d,f). Pattern 9 (1 μm wells) had significantly larger soma areas than all other substrates showing *p* < 0.0001 with most topographies. Thus, Pattern 9 shows a specialized effect on hippocampal mNPCs generating very large astrocytes. Pattern 1 (2 μm gratings with perpendicular 250 nm gratings) generated astrocytes with the smallest soma areas (7.6 ± 0.6 × 10^2^ μm^2^) which were still significantly larger than cortical mNPC-derived astrocytes on the same pattern (Figure 6e). 

Thus, the inherent differences between cortical and hippocampal mNPC-derived astrocytes discussed earlier were reflected in all the morphological parameters quantified. While cortical mNPC-derived astrocytes possessed small soma areas, they showed a number of short, clearly-defined projections with multiple branches per projection. Hippocampal mNPC-derived astrocytes possess larger soma areas with few but long projections with almost no branches.

### 2.6. The Correlation of Morphology and Differentiation Efficiency Is Dependent on the Type of NPCs

**Figure 7:**
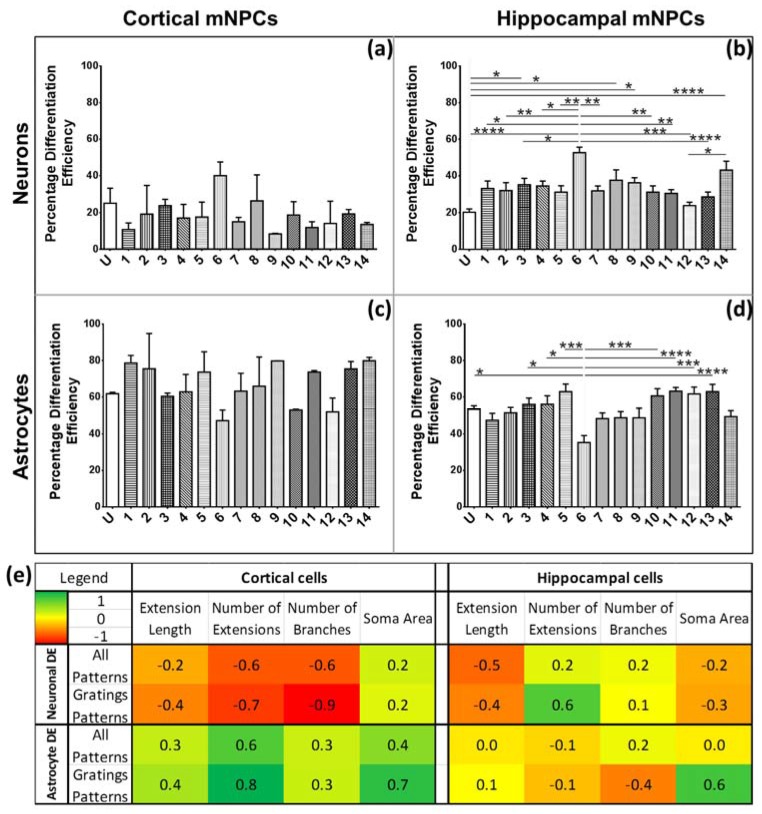
Cortical and hippocampal mNPC neuron differentiation efficiencies and their correlations with morphological parameters. Graphs depict neuronal differentiation efficiency for (**a**) cortical mNPCs, (**b**) hippocampal mNPCs, and astrocyte differentiation efficiency for (**c**) cortical mNPCs, and (**d**) hippocampal mNPCs. One-way ANOVA: * *p* < 0.05, ** *p* < 0.01, *** *p* < 0.001, **** *p* < 0.0001. All data is represented as data ± SEM. (**e**) Heat map showing the correlation coefficients of neuronal and astrocyte differentiation on the 14 patterns with each measured morphological parameter on the corresponding patterns. Correlation coefficients were also calculated separately for cells differentiated on gratings patterns. Colour indicates the intensity of correlation as shown in the legend in the top left corner. Left: cortical cells. Right: hippocampal cells. *N* = 3 biological replicates.

The differentiation efficiencies for hippocampal and cortical NPCs to produce neurons and astrocytes were observed to vary with topography (Figure 7a–d), as has also been reported in the literature [4,10,11,18]. For hippocampal cells, differentiation efficiency was the least on the unpatterned control (significantly lower than Patterns 3, 6, 9, and 14). In line with the earlier study of hippocampal differentiation on an array of topographies [10], Pattern 6 (2 μm gratings with 2 μm spacing and 2 μm height) generated neurons most efficiently (52.68 ± 2.96%), with a significantly higher differentiation efficiency than most of the patterns studied. In contrast with hippocampal mNPC neuronal differentiation, astrocyte differentiation was significantly less (35.31 ± 3.81%) on Pattern 6 (2 μm gratings with 2 μm spacing and 2 μm height) in comparison with most patterns (unpatterned control, Patterns 3, 4, 5, 10, 11, 12, and 13) (Figure 7d). This result is again in agreement with previous reports of 2 μm gratings showing high neuron to astrocyte ratios during differentiation of NPCs [11].

In this context, it was of interest to understand the possible morphological parameters that correlated with higher or lower differentiation efficiencies. Since anisotropic topographies, such as gratings, have been shown to result in a higher neuron to astrocyte production ratio [10,11,18], the relation between morphology and differentiation on gratings was also calculated separately. 

It was observed that the correlation to differentiation efficiency was largely dependent on the source of NPCs (Figure 7e). Cortical mNPC-derived neurons showed a negative correlation (correlation coefficient of −0.6) between the differentiation efficiency and the number of extensions expressed on the cell. The opposite relation was observed for hippocampal mNPC-derived neurons (correlation coefficient of 0.2). These trends were even more pronounced on gratings patterns with cortical and hippocampal mNPC-derived neurons showing correlation coefficients of −0.7 and 0.6, respectively. Therefore, cortical cells showed an increase in neuronal differentiation with an increase in the number of extensions per neuron, contrary to the observed effects on hippocampal cells. 

Correlations between astrocyte differentiation and morphology were also observed to be dependent on the type of mNPCs considered. The astrocyte differentiation efficiency of cortical mNPCs showed a positive correlation (correlation coefficient of 0.6) with the number of extensions present on the cells. On the other hand, the astrocyte differentiation efficiency of hippocampal mNPCs was independent (correlation coefficient of −0.1) of this same morphological trait. The neuronal differentiation efficiency of cortical mNPCs was also correlated with a decrease in the number of branches expressed by neurites (correlation coefficient of −0.6). Furthermore, interesting trends emerged when differentiation on gratings (Patterns 1–8 only) was correlated with morphology on gratings. The correlation of cortical mNPC astrocyte differentiation with the number of extensions was enhanced on grating substrates (correlation coefficient of 0.8 on Patterns 1–8 versus 0.6 on all topographies). The soma area for both types of mNPC-derived astrocytes was consistently correlated with the differentiation efficiency (correlation coefficients of 0.7 for cortical and 0.6 for hippocampal mNPC-derived astrocytes). Thus, the types of gratings which produce larger soma areas are associated with an increase in the derivation of astrocytes. In addition, cortical mNPCs differentiated on gratings substrates showed a strong negative correlation with the number of branches expressed by neurites (correlation coefficient of −0.9). This negative correlation could explain why cortical neurons differentiated on gratings often display less branching.

In summary, the correlations between morphology and differentiation were influenced by the origins of the NPCs. Most strikingly, grating topographies were observed to enhance correlations between morphology and differentiation for both types of mNPCs.

## 3. Discussion

The species of origin, the developmental stage of the progenitor (embryonic versus post-natal or adult), and the location from which progenitors are isolated from the nervous system have been shown in the literature to have a large impact in the outcome of neuronal and astrocyte differentiation. Neural progenitors have been shown to have varying capabilities for proliferation, differentiation, and morphology across different mammals [20,21,22]. Specifically, neural stem cells have been systematically studied to be affected by developmental stage, brain region, and time in culture for their proliferation and differentiation. It has been reported that there is a developmental stage dependent decrease in the neurogenecity of cells as the population becomes more glial. Although not quantified, region-dependent differences in neuronal and astroglial morphology, independent from the donor tissue age, were shown to have region dependent intrinsic features of neuronal and glial progenitors [12]. Seaberg et al. observed that when postnatal neural precursors from different locations of the brain (namely, the cerebral cortex, the lateral striatum, and optic nerve) were tested with varying days of isolation from the animal, the neurogenecity was shown to be different, depending on the location and age of the progenitors [13]. Moreover, the neurogenic capacity of the cells was shown to be an intrinsic property, despite the use of different medium compositions [13]. These studies strongly suggest that there are intrinsic differences in differentiation potential between the different types of NPCs. 

Similarly, astrocytes have been reported to show heterogeneity that closely reflects the phenotypic regions of the brain defined by neurons [23]. Astrocyte morphology is subject to factors such as their location in the CNS and the number of days that the cells have undergone morphological maturation [22]. Postnatal hippocampal astrocytes have been categorized as protoplasmic astrocytes, which display ‘spongiform’ morphology. In addition, plasmodial astrocytes in the hippocampus possess a spongiform morphology [24]. Over the course of morphological maturation, astrocytes undergo specific changes; their filopodial processes elongate and larger processes extend from their bodies. Beyond the first three weeks of maturation, astrocytes undergo a change in the heterogeneity of morphology itself, which drastically reduces as the cells mature further. The process is completed after the stabilization of competitive overlapping cell domains [25]. Astrocytes developing in a large and diverse region of the brain, such as the cortex, display a much more heterogeneous combination of morphologically defined astrocyte categories [26]. This could explain the spongiform versus astral morphology that was observed in the hippocampal versus the cortical mNPC-derived astrocytes on the topographical substrates in the present study. The morphological differences between cortical and hippocampal mNPCs derived neurons and astrocytes when exposed to the same topographies are, thus, inherent, as shown in the literature. 

Notably, the differences in morphology could also be contributed by other factors. In the current study, hippocampal NPCs were isolated from the hippocampi of five day old mice. On the contrary, cortical NPCs were isolated from the whole cortices of 13-day old mouse embryos at the peak of their neurogenetic phase and were grown in the ideal media required for differentiation. While hippocampal cells require the use of two different media for the generation of neurons, cortical cells were differentiated in a single differentiation medium (Figure S2). In addition, cortical cells required a period of five days for the expression of the TUJ1 marker, whereas neuronal differentiation of hippocampal cells required 14 days. Thus, the ideal cell culture conditions were chosen for each cell type. As a reference for comparison, morphological measurements on topographical substrates were accompanied with measurements of cells on an unpatterned control. The control allowed the observation of the intrinsic differences between the two cell types which were isolated from different regions of the brain, at different developmental stages, and cultured with different media. Together, the differences in the age of the mice, the region of the brain, and the culture conditions required could also result in the differences observed in the differentiation efficiency.

In this study, topography was used as a tool to influence NPC differentiation and establishment of cell morphology. Four morphological parameters were studied in neurons and astrocytes differentiated from cortical and hippocampal mNPCs. 

Firstly, the length of the longest extension on each cell and the number of branches on each extension were measured. A change in neurite length has been shown to be associated with neural diseases such as Rett syndrome [15] and Parkinson’s disease [14]. In addition, neurite length has been shown to be influenced by topography. In particular, differentiating hippocampal mNPCs have been reported to show a size dependent relation between neurite extension and grating depth. Neurites were observed to extend across shallow gratings where it was possible for filopodia to adhere to the bottom of the groove. In the case where grating depth was comparable to grating width, the extending neurite was no longer able to sense the presence of the substrate and did not continue extension. This led to the alignment of neurites on 1:1 aspect ratio microgrooves as reported previously [18]. A similar observation was made in the current study (Figure S1h), where neurites were observed to extend into micron scale gratings. In addition, differentiated cortical NPCs displayed shorter extensions in comparison to hippocampal cells (Figure 1). Hippocampal neurons specifically showed an increase in neurite length on pillars. The observation of longer neurites on isotropic topography is in contrast with the vastly reported elongation effects of anisotropic structures such as grating topographies on neuronal cells [11]. However, previous studies have also reported that rat hippocampal neurons on pillar topographies have increasing extension lengths with increasing width. It was found that extension lengths were the longest on pillars with a 2 μm width [27]. In addition, E18.5 mouse hippocampal neurons have been shown to have faster neurite elongation on nanopillars with specifically 500 nm spacing. Neurites were reported to extend on top of the pillars, which provide optimally spaced points for substrate adhesion [28]. Hippocampal cells also possessed fewer branches on both neurons and astrocytes. The branching is independent of the expression of projections, as was observed in the quantification of the average number of projections and branches per projection (Figure 4 and Figure 5). Thus, hippocampal mNPC-derived astrocytes not only expressed few projections from the cell center, but also a very small degree of branching on the cells that did possess projections. These properties seem to be unique to hippocampal cells. 

Secondly, soma areas of both cell types were measured on each of the topographies. Here, hippocampal mNPC-derived astrocytes were observed to possess much larger soma areas than other hippocampal NPC derived neurons, as well as neurons and astrocytes derived from cortical mNPCs. In addition, the area of these cells did not show much variation between topographies. These morphological attributes were also observed in immunofluorescence images (Figure 1b) and may be an intrinsic trait of the cells as described earlier. The increase of soma size in association with topography would be an interesting tool to observe cells in disease states and developmental states. Soma sizes have been shown to drastically increase during human adolescence [29]. Soma sizes also tend to be altered in diseases such as Costello Syndrome [30], Rett Syndrome [19], and depression [31]. Although nanogrooved substrates have been reported to reduce soma sizes during the production of dopaminergic neurons [32], the effect of topography has been largely unexplored in disease cells. It, would be interesting to observe patient iPSC derived neurons and astrocytes on topography because they have been shown to have significantly different soma sizes.

**Figure 8:**
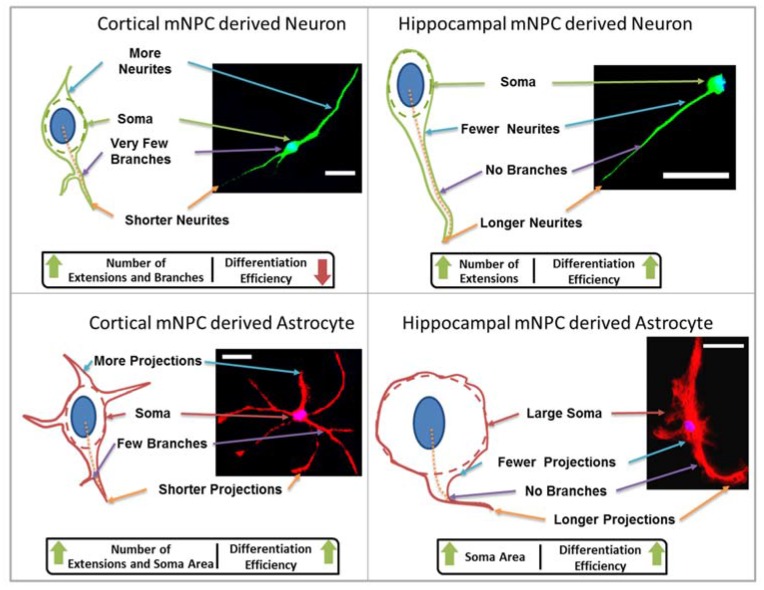
Typical cell morphologies and correlations to differentiation. Typical morphological characteristics of differentiated cortical and hippocampal cells are illustrated alongside immunofluorescence images of typical cells (green: neurons, red: astrocytes, blue: nuclei. Scale bar = 20 μm). Labels indicate relative morphological peculiarities. Relations between morphological parameters and efficiency of differentiation into specific cell types are indicated in boxes below the illustrations.

The characteristic morphological traits for cortical and hippocampal mNPC-derived neurons and astrocytes have been identified in the current study. These traits were expressed by cells regardless of topographical influence on cell morphology and have been summarized in Figure 8. There is a range of morphological changes, as well as various differentiation efficiencies caused by the influence of an array of different topographies. This allowed for the study of the correlation between cell shape and cell fate. Different trends were observed for the two types of differentiated mNPCs. For example, while cortical neural progenitor derived neurons showed a negative correlation between differentiation efficiency and the number of extensions from the cell center, an opposite relation was observed for hippocampal neural progenitor derived neurons. However, astrocyte soma areas showed similar correlations with differentiation in both types of NPCs. Notably, gratings topography was shown to enhance the correlation between cell shape and differentiation in all cell types. The correlations have been summarized in Figure 8. Thus, gratings that produce morphological effects, such as an increase in the number of extensions, were associated with an increased or decreased production of neurons, depending on the type of neural progenitor. The enhancement of neuronal differentiation by gratings substrates has been extensively reported [4,10,18,33]. Our findings suggest that this enhancement could be attributed to the morphological effects of gratings topography on neural progenitor cells. Therefore, gratings may promote the morphological characteristics necessary for enhanced production of neurons. However, the morphological characteristics associated with increased neuronal differentiation are largely dependent on the source of neural progenitor cells.

The differentiation of neural progenitors on topography has been reported in numerous studies. Similarly, neuronal morphology has also been established as a field. The difference between neural cells from different regions of the brain has been established in literature. The current study quantitatively compares the differentiation and morphology of neural progenitors from the hippocampus and cortex on an array of topographies. Moreover, a correlation between cell shape and differentiation was established. However, further studies are necessary to determine a causative relation between neuron production and cell shape. Grating substrates prove to be the ideal candidates for these studies. 

## 4. Materials and Methods 

### 4.1. Fabrication and Replication of PDMS Substrates

The multi-architecture (MARC) chip is an array of multiple nano- and micro-topographical patterns. To assemble the MARC chip, the fabrication of individual patterns of 2 mm × 2 mm in size (large enough to allow for statistical analysis of cells) via nano-imprinting lithography was completed. Subsequently, these individual patterns were arranged onto a single silicon chip pre-coated with poly-dimethylsiloxane (PDMS, Sylgard 184 polymer, Dow Corning, Auburn, MI, USA) as a bonding material. Based on how the individual patterns are cut, they may either be circular [10] or square-shaped. The versatility of the MARC chip lies in the choice of patterns one can arrange onto each chip, making it possible to combine different or repeating patterns. The MARC chip can also be customized to be of a different shape and size to accommodate different applications, such as microfluidic systems [25]. Replicas of the MARC chip were generated by soft lithography using PDMS and replication was verified by scanning electron microscopy (JEOL JSM 6010 LV, JEOL, Tokyo, Japan) by sputter coating the samples with platinum at 30 mA for 30 s and observing them at various magnifications at high vacuum (Figure S1a–h). Silicon molds of 44-pattern and 18-pattern MARC chips were used for testing multiple topographies at once, along with an unpatterned substrate as a control. Table 1 shows the topographical patterns that were studied.

For the generation of replicas, PDMS was mixed in a 10:1 ratio with the curing agent and placed under vacuum for removal of air bubbles. The mixture was then poured onto the MARC chip molds. After additional removal of air under vacuum for 30 min, the PDMS was cured at 70 °C for 2 h. Replicas were generated upon demolding. The PDMS replicas were cut according to the wells of the culture plates. To remove hydrophobicity, replicas were treated with oxygen-plasma (Harrick Plasma, Ithaca, NY, USA) for 1 min at 30 W. For sterilization, samples were washed with 70% ethanol and placed under ultraviolet light for 20 min. To allow attachment of laminin, replicas were coated overnight with poly-L-ornithine solution (33 μg/mL) in sterile deionized water (Sigma-Aldrich, St. Louis, MO, USA). After two washes with sterile deionized water, they were coated overnight with 20 μg/mL mouse laminin in Dulbecco’s modified Eagle medium (DMEM)/nutrient mixture F12 (Biological Industries, Cromwell, CT, USA) in a 1:1 ratio (1:1 DMEM/F12) (Life Technologies, Waltham, MA, USA) to carry out differentiation of mNPCs. 

### 4.2. Culture of Murine Neural Progenitor Cells (mNPCs)

Hippocampal neural progenitor cells were isolated from the hippocampus of five day old mice [10]. Cortical NPCs were isolated from the whole cortex of 13 day old mouse embryos (E13). Each NPC was established as a monolayer culture, as previously described for hippocampal NPCs [34] Both mNPCs were a gift from Dr. Eyleen Goh’s group (Duke-NUS Graduate Medical School, Singapore). The cells were cultured in neural progenitor expansion medium, which contains 1:1 DMEM/F12Dulbecco’s modified Eagle medium (DMEM)/nutrient mixture F12 (Biological Industries, Cromwell, CT, USA) in a 1:1 ratio supplemented with 1× N2 supplement (Life Technologies, Waltham, MA, USA), and 1× penicillin-streptomycin (Caisson Biotech, Austin, TX, USA). This medium was supplemented daily with 20 ng/mL basic fibroblast growth factor (bFGF) (Life Technologies, Waltham, MA, USA) and 20 ng/mL epidermal growth factor (EGF) (R&D Systems, Minneapolis, MN, USA). It was changed every two days. Cells were passaged at 80% confluence with Accutase (Innovative Cell Technologies Inc., San Diego, CA, USA). After centrifugation of the cell suspension at 1000 rpm for 5 min, cells were split into a 1:4 ratio and transferred to plates coated with laminin. To maintain phenotypic integrity, cells were cultured to a maximum passage number of 25.

### 4.3. Differentiation of Cortical mNPCs

For cortical mNPCs, cells were seeded at a density of 5 × 10^4^ cells per cm^2^ on PDMS replicas with neuronal differentiation medium consisting of DMEM/F12 with 1× N2 supplement, 1× B27 medium, and 1× penicillin-streptomycin solution, without any growth factors. Every two days, half of the medium volume was refreshed. Samples were fixed on day 5 for further analysis (Figure S2).

### 4.4. Differentiation of Hippocampal mNPCs

Differentiation of hippocampal mNPCs was carried out at a seeding density of 7.5 × 10^3^ cells per cm^2^ on PDMS replicas as described in Moe et al. [10]. Briefly, the differentiation period was divided into two phases: the induction phase and the maturation phase (Figure S2). The neural induction medium induces neurogenesis by withdrawal of the growth factors necessary to maintain the cells as progenitors. The induction medium consists of DMEM/F12 with 1× N2 supplement, 1× B27 medium, 5 ng/mL FGF2 (Life Technologies, Waltham, MA, USA), laminin (1 μg/mL), and 1× penicillin-streptomycin solution. Every two days, half of the medium volume was refreshed. On day 7, the induction medium was replaced with the maturation medium. The maturation medium consisted of a 1:1 ratio of Neurobasal (Life Technologies, Waltham, MA, USA) and DMEM/F12 with 1× B27 supplement (Life Technologies, Waltham, MA, USA), 0.25× N2 supplement, and 1× penicillin-streptomycin. Every two days, half of the medium volume was refreshed. On day 14 of culture, samples were fixed for further analysis. Cells cultured in this manner from the dataset described in Moe et al. [10] were analysed in this study.

### 4.5. Immunofluorescence Staining of Class III β-Tubulin and Glial Fibrillary Acidic Protein

Cells were fixed for 20 min with 4% paraformaldehyde, and then blocked for 60 min with 10% goat serum (Life Technologies, Waltham, MA, USA) and 0.1% Triton X-100 in PBS. Primary antibodies against Class III β-Tubulin (TUJ1) (1:600 rabbit polyclonal AB_262133, Sigma, St. Louis, MO, USA) were used to mark neuronal cells. Antibodies against and glial fibrillary acidic protein (GFAP) (1:600 mouse monoclonal AB_2109815, Millipore, Billerica, MA, USA) were used to mark astrocytes. The antibodies were diluted in 1% goat serum and incubated overnight at 4 °C. This was followed by 1 h of incubation with the appropriate secondary antibodies at room temperature. The secondary antibodies used were goat anti-rabbit Alexa Fluor 488 (polyclonal AB_2576217, Molecular Probes, Eugene, OR, USA), and goat anti-mouse Alexa Fluor 546 (polyclonal AB_144695, Molecular Probes, Eugene, OR, USA) at the concentration of 1:500. Cells were counterstained with 4′,6-diamidino-2-phenylindole (DAPI) (Sigma-Aldrich, St. Louis, MO, USA) for 20 min before mounting with Prolong Gold antifade mounting media (Life Technologies, Waltham, MA, USA). The images were taken with an inverted fluorescence DMIRM microscope (Leica, Wetzlar, Germany), confocal microscope SP5 (Leica, Wetzlar, Germany), and confocal microscope Nikon A1r. For comparison with differentiated hippocampal neural progenitor cells, images from Moe et al. [10] were analysed. Uniform brightness and contrast (histogram stretch) adjustments were made for each panel of immunofluorescence images represented in Figure 1 using ImageJ.

### 4.6. Morphological Parameter Measurements

The morphological parameters of the astrocytes and neurons were measured from the GFAP and TUJ1 populations. respectively. For image analysis, ImageJ (version 1.46r) and a custom-made program in MATLAB (version R2013a, MathWorks, Natick, MA, USA) were used. The MATLAB program had a graphical user interface for the tracing of neurite extensions (Figure S3) that was designed to rapidly process large data sets. The algorithms were semi-automated and relied on initial user input regarding the rough localization of cell features. The tracing was then iteratively refined using active contour algorithms [35]. For each cell processed, the program automatically created and saved a dataset which described the topology and geometry of the neuron, using the adjacency matrix and other means from graph theory [36]. From this dataset, the following morphological parameters were measured (Figure 2):

- Number of extensions: Number of extensions from the cell center (i.e., the number of neurites per neuron and number of projections per astrocyte).
- Number of branches per extension: Total number of branches divided by total number of extensions per cell. A branch was defined as a secondary protrusion from an extension originating from the cell center. Thus, each extension could possess multiple branches. When an extension from the cell center displayed no secondary protrusion, the neurite was counted as possessing zero branches.
- Extension Length: The length of the longest extension from the cell body.
- Soma area: Area of the cell body excluding extensions. This was identified by ellipse approximation using a function of ImageJ.

All images used for quantification were TIFF images. For quantification of the morphology of differentiated cortical mNPCs, maximum intensity projections of confocal image stacks were taken. For the measurement of the number of extensions, extension length, and the number of branches of both, images were adjusted in brightness and contrast individually for maximization of signal recognition by the MATLAB active contour algorithms and to ensure visibility of as many cells as possible. The algorithm was semi-automated such that the cell center and specific points along the extensions were manually defined to limit the program to the cell space from which individual cell skeletons were created (Figure S3). The accuracy of the active contour algorithm was manually adjusted as necessary. This was achieved by adjusting parameters, such as point spacing, smoothing, fitting edge, and adjusting image and mechanical forces, such as contribution, tension, and bending. The iteration time was also set to maximize signal recognition by the software. Taken together, the presence of noise did not influence the parameter measurement. 

For the measurement of the soma area, ImageJ software was used. Images were individually adjusted for brightness and contrast to ensure the visibility of cell somas; then they were ”despecked”, threshold-adjusted for noise reduction, and converted to binary. The “watershed” function was used for the preparation of images for calculation of the soma area through the “fit ellipse” function of the “analyse particles” macro.

Prism 6.0 (Graphpad Software Inc., La Jolla, CA, USA) was used for data analysis. Data were presented as means and the standard error of means. For the comparison of data on different topographies, one-way analysis of variance (ANOVA) was performed, followed by a Tukey’s multiple comparisons test. For the comparison of data between cortical and hippocampal mNPCs, two-way ANOVA was performed, followed by a Sidak’s multiple comparisons test. *p* values less than 0.05 were considered statistically significant. The number of cells examined for each replicate ranged from 30–150 cells. The total number of cells (differentiated neurons, astrocytes and undifferentiated cells) measured for each of the topographies was as follows: Cortical Cells- U: 583, 1:333, 2:342, 3:363, 4:269, 5:378, 6:131, 7:149, 8:167, 9:237, 10:149, 11:270, 12:158, 13:782, 14:716; Hippocampal cells- U: 1144, 1:911, 2:754, 3:738, 4:1035, 5:1109, 6:579, 7:1046, 8:436, 9:728, 10:889, 11:920, 12:812, 13:1075, 14:689.

Differentiation efficiency (DE) was calculated as the percentage of cells expressing TUJ1 (neuronal differentiation efficiency) or GFAP (astrocyte differentiation efficiency). Hippocampal mNPC differentiation efficiency was calculated from images belonging to the dataset described in Moe et al. [10], which was performed in a similar manner, with permission of John Wiley and Sons.

For further analysis, correlation coefficients were calculated by comparing measured values of each morphological parameter on all topographies with the differentiation efficiencies of all topographies. For the gratings pattern subset, DE values and morphological measurements were selected from cells grown on grating topographies. Neuronal DE was compared exclusively with neuronal morphological parameters. Similarly, astrocyte correlations were calculated separately. In addition, correlation coefficients were not calculated between types of neural progenitor cells but rather within the same subset.

## 5. Conclusions

The current study revealed that cell source determines varied responses to topographical cues both in differentiation and morphology. Pillar and well topographies were shown to have an impact on cell extension lengths and soma areas. The degree to which neural progenitor cells were influenced by topography was dependent on the source of the neural progenitor. Neural progenitor cells from the hippocampus and cortex produced neurons and astrocytes with different efficiencies. Thus, the inherent differences in the cell source were also apparent in the possible relationship between morphology and differentiation on an array of topographies. Interestingly, grating topographies established stronger connections between cell morphology and differentiation than other topographies. 

The relation between differentiation efficiency and cell shape continues to be complex. This study of contact guidance mediated topographical effects on neuronal morphology has contributed to our understanding of how gratings topography continues to be influential in shaping neuronal shape and cell fate.

## Figures and Tables

**Table 1:** Topographical patterns studied.

Category			No.	Pattern Description
Gratings	Micron range	Hierarchy	1	2 μm gratings × 2 μm spacing and perpendicular 250 nm grating × 250 nm spacing overlay on crests
			2	2 μm gratings × 2 μm spacing and parallel 250 nm grating × 250 nm spacing overlay on crests
			3	2 μm gratings × 2 μm spacing and 250 nm dimples overlay
			4	1 μm gratings × 2 μm spacing × 120 nm height
			5	2 μm gratings × 1 μm spacing × 80 nm height
		1:1:1 Aspect Ratio	6	2 μm gratings × 2 μm spacing × 2 μm height
	Nano range		7	250 nm gratings × 250 nm spacing × 250 nm height
			8	250 nm gratings × 250 nm spacing × 150 nm height
Wells	Micron range		9	1 μm diameter wells with 6.5 μm pitch and 1 μm depth
Pillars	Micron range		10	2 μm diameter pillars with 12 μm pitch and 2 μm height
	Nano range		11	500 nm diameter pillars with 10 μm pitch and 500 nm height
			12	130 nm diameter, 400 nm pitch, 230 nm height
Lenses	Micron range		13	Microlenses: 1 µm pitch, 0.3 µm sag (convex)
			14	1.8 μm diameter with 2 μm pitch and 0.7 μm sag (convex)

## Supplementary Materials

The following are available online at www.mdpi.com/2079-4983/8/3/35/s1, Figure S1. Scanning electron micrographs of select topographies, a typical neuron on gratings, and representative phase contrast images of differentiated cells. Figure S2. Cell culture timeline for hippocampal mNPCs and cortical mNPCs. Figure S3. User interface of MATLAB algorithm and representative cell trace used for the measurement of morphological parameters.
