# Supplementary material for: Correlation and Comparison of Cortical and Hippocampal Neural Progenitor Morphology and Differentiation through the Use of Micro- and Nano-Topographies
Source: J Funct Biomater. 2017 Aug 12;8(3):35. doi: 10.3390/jfb8030035 (PMC5618286; doi:10.3390/jfb8030035)
Supplement: Supplementary file 1 [file jfb-08-00035-s001.pdf]

Journal of
*Functional
Biomaterials*

Article

Correlation and Comparison of Cortical and Hippocampal Neural Progenitor Morphology and Differentiation through the Use of Micro- and Nano-Topographies

Sharvari Sathe [1], Xiang Quan Chan [2], Jing Jin [2], Erik Bernitt [3], Hans-Günther Döbereiner [1,3], Evelyn K.F. Yim [1,2,4,5,*]

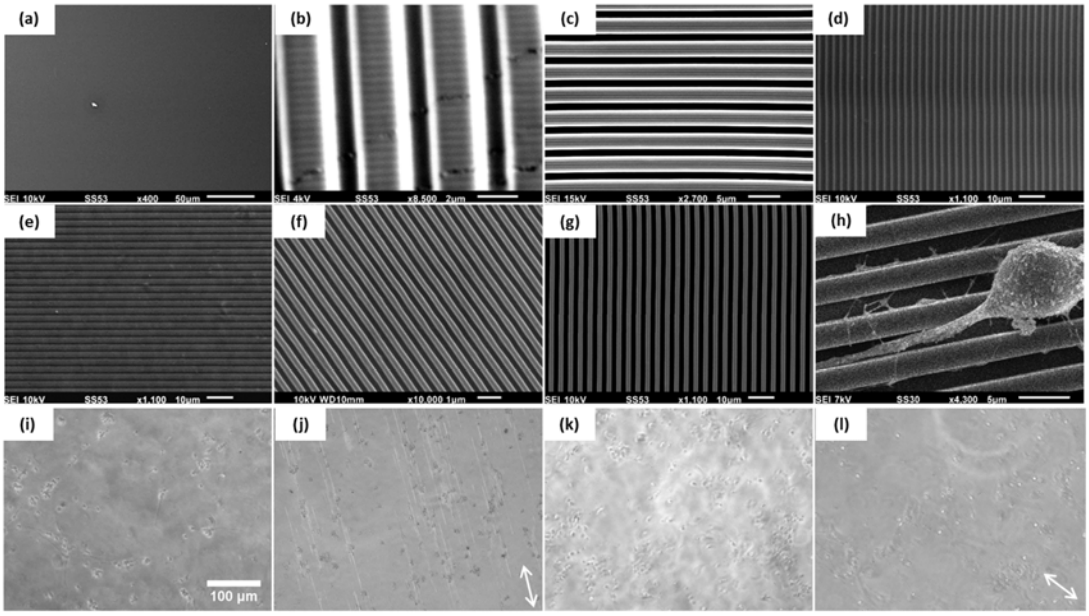

Figure S1. Scanning electron micrographs of select topographies, a typical neuron on gratings, and representative phase contrast images of differentiated cells. (**a**) Unpatterned substrate, Scale bar = 50µm; (**b**) 2 µm gratings × 2 µm spacing and perpendicular 250 nm grating × 250 nm spacing, Scale bar = 2 µm; (**c**) 2 µm gratings × 2 µm spacing and parallel 250 nm grating × 250 nm spacing, Scale bar = 5 µm; (**d**) 1 µm gratings × 2 µm spacing × 120 nm height, Scale bar = 10 µm; (**e**) 2 µm gratings × 1 µm spacing × 80 nm height, Scale bar = 5µm; (**f**) 250 nm gratings × 250 nm spacing × 250 nm height, Scale bar = 1µm; (**g**) 2 µm gratings × 2 µm spacing × 2 µm height , Scale bar = 10 µm; (**h**) Cortical mNPC-derived neuron on 2 µm gratings with 2 µm spacing and 2 µm height, Scale bar = 5 µm. Representative phase contrast images of differentiated cortical mNPCs on an (**i**) unpatterned substrate and (**j**) 2 µm gratings with 2 µm spacing and 2 µm height; and hippocampal mNPCs on an (**k**) unpatterned substrate and (**l**) 2 µm gratings with 2 µm spacing and 2 µm height, Scale bar = 100µm, white arrows indicate grating direction.

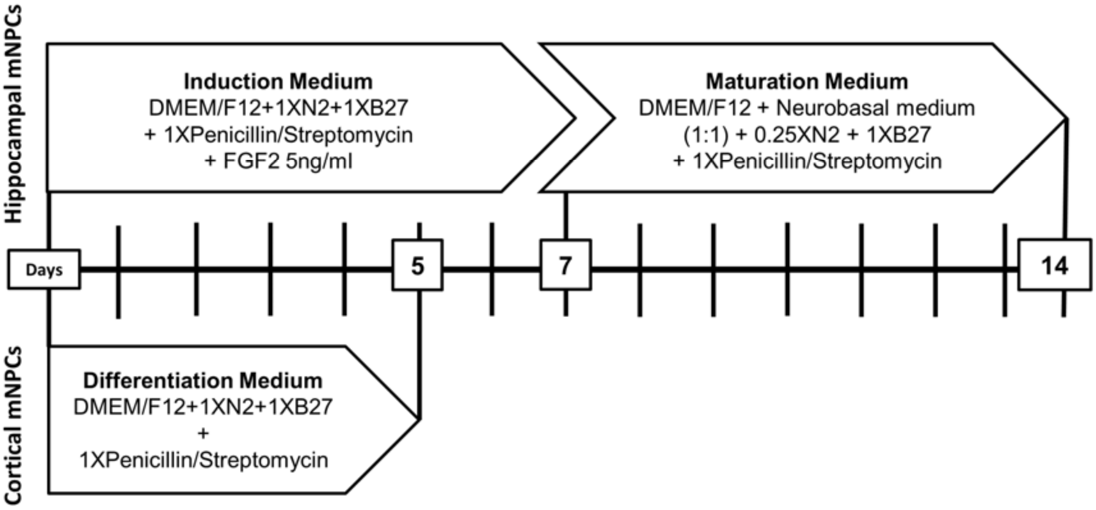

Figure S2. Cell culture timeline for hippocampal mNPCs and cortical mNPCs. Hippocampal cell culture involves the use of induction medium for the first 7 days followed by a change to maturation medium up to day 14 in culture. Cortical cells were differentiated for 5 days in differentiation medium.

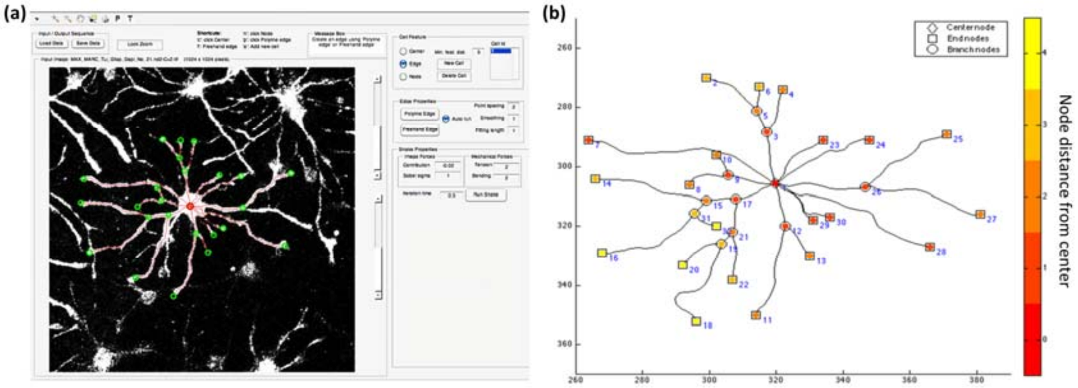

Figure S3. User interface of MATLAB algorithm and representative cell trace used for the measurement of morphological parameters. (**a**) A semi-automated algorithm was used to trace cell morphology with manual corrections. Contour recognition algorithms were adjusted for best fit; (**b**) Nodes were used to identify the cell center, a branch point or the end of an extension and are indicated in the legend. The cell traces were used to measure the length of the longest extension, the number of extensions and the number of branches per extension on each cell.

© 2017 by the authors. Submitted for possible open access publication under the terms and conditions of the Creative Commons Attribution (CC BY) license (http://creativecommons.org/licenses/by/4.0/).
